# Supplementary material for: Fluorescent fatty acid conjugates for live cell imaging of peroxisomes
Source: Nat Commun. 2024 May 21;15:4314. doi: 10.1038/s41467-024-48679-2 (PMC11109271; doi:10.1038/s41467-024-48679-2)

Supplementary Information

Figure Legends

Supplementary Figure 1 Staining of cellular organelles with fluorescent conjugates

(a-c) Confocal microscopy of live HEK293T cells stained with (a) BODIPY (magenta) and BODIPY methyl ester (Cell Trace, cyan) (1µg/ml), (b) BODIPY-Cholesterol(cyan) and Nile Red (magenta), (c) BODIPY-C12 (cyan) and NIR633 (1µg/ml, magenta) for 10 min. Nuclei were stained with Hoechst (10µg/ml). Representative intensity profiles are shown, scale bars – 5µm.

(d) Cumulative cell intensity profile for live HEK293T cell expressing GFP-SKL (magenta), stained with BODIPY-C12 (1µg/ml, cyan) for 10 min. Refer to Figure 1b (Cell with an inset).

(e) Confocal microscopy of live U2OS cells expressing GFP-SKL(magenta), stained with BODIPY-C12 (1µg/ml, cyan) for 10 min. Nuclei were stained with Hoechst (10µg/ml). A representative intensity profile is shown, scale bars - 5 and 1µm.

(f) Confocal microscopy of fixed CRISPR/Cas9 PMP70 KO and WT HEK293T cells stained with PMP70 and PEX14 antibodies. Nuclei were stained with Hoechst (10µg/ml), scale bar - 5µm.

(g) Confocal microscopy of live control, PEX3 null patient-derived fibroblasts stained with PeroxiSPY555 and BODIPY-C12 (1µM) for 15min.

Supplementary Figure 2 Probe synthesis

(a) Schematic of chemical synthesis

(b) SiR-C16 1H NMR spectrum in MeOD

(b) SiR-C18 1H NMR spectrum in CDCl3

Supplementary Figure 3 Probe synthesis

(a) Peroxy-SPY650 1H NMR spectrum in MeOD

(b) MaP555-azide 1H NMR spectrum in MeOD

Supplementary Figure 4 Probe synthesis

(a) 555-C18 1H NMR spectrum in MeOD

(b) PeroxySPY555 1H NMR spectrum in MeOD

Supplementary Figure 5 Probe comparison in human cells

(a-b) Confocal microscopy of live WT and PEX19 KO HEK293T cells stained with PeroxiSPY650 (cyan), PeroxiSPY555 (magenta), MaP555-C18 (magenta), SiR-C16 (cyan), SiR-C18 (cyan), and BODIPY-C12 (1µM, yellow). Quantification shows the ratio of peroxisome to cytoplasm fluorescence intensity of BODIPY-C12, mean± SEM, ** - $p<0.01$, **** - $p<0.0001$, N=3 pooled from >1000 peroxisomes for each sample, one-way Anova.

(c) Confocal microscopy of live WT, PEX5 KO, and complemented PEX5 KO PEX5L HEK293T cells, stained with PeroxiSPY555 (1µM) for the indicated amounts of time, scale bar - 10µm.

(d-e) Quantification of confocal microscopy of live WT and PEX5 KO HEK293T cells, stained with PeroxiSPY650 or BODIPY-C12 (1(cyan), 2(black), and 0.5µM(black)) for the indicated amounts of time, scale bar - 10µm. Graphs show the ratio of cells with identifiable peroxisomes, N=3 pooled from at least 300 cells for each time point.

Supplementary Figure 6 Effect of probes on cellular viability

(a) Cell toxicity (LDH release) assay, showing % of LDH release in live HEK293T WR and PEX19 KO cells incubated with indicated amounts of dyes for 24 hours (PeroxiSPY650, PeroxiSPY555, BODIPY-C12). Quantification shows the % of LDH release, mean± SEM, ** - p<0.01, N=3, one-way Anova.

(b-c) Cell growth assay showing HEK293T WT cells incubated with indicated amounts of dyes or DMSO (cyan) for 48 hours (PeroxiSPY650 (magenta), PeroxiSPY555(black), MaP555-C18(black), SiR-C16 (magenta), SiR-C18 (magenta)). Quantification shows cell counts at indicated times, mean± SEM, ** - p<0.01, N=6, Kruskal-Wallis test.

Supplementary Figure 7 Visualizing peroxisomes in a live Zebrafish embryo

(a) Confocal microscopy of live Zebrafish embryo on stage 18hpf, stained with BODIPY-C12 (1µg/ml) for 10min, and mounted in a soft agar pad prior to imaging. Scale bars – 100, 10, and 1µm. TD – transmission detector.

(b) Confocal microscopy of live Zebrafish embryos (18hpf) cells injected with mRNA encoding GFP or GFP-SKL. Scale bar - 10µm.

(c-d) Confocal microscopy of live Zebrafish embryos (18hpf) cells injected with mRNA encoding GFP or GFP-SKL(magenta), stained with BODIPY-C12 (1µg/ml, cyan) for 10min, and mounted in a soft agar pad prior to imaging. Scale bars - 5 and 1µm. (c) Intensity profile through single peroxisome. Refer to Figure S8B for a cumulative profile. PCC=0.547.

(e-f) Confocal microscopy of live Zebrafish embryos (16hpf) injected with mRNA encoding GFP or GFP-SKL(magenta), stained with PeroxiSPY650 (1µM, cyan) for 10min and 20min, and mounted in a soft agar pad prior to imaging. Scale bars - 5 and 1µm. Representative cumulative intensity profiles are shown, PCC (10min) =0.355, PCC (20min) =0.574.

Supplementary Figure 8 Visualizing peroxisomes in plants and Zebrafish

(a) Confocal microscopy of live Zebrafish embryo, stained with BODIPY-C12 (1µg/ml) mixed with bovine serum albumin (BSA) for 10min, scale bar - 100µm.

(b-d) Fluorescence intensity profiles in live Zebrafish tissues during BODIPY-C12 (1µg/ml, cyan) or PeroxiSPY650 (1µM, cyan) staining, peroxisomes are visualized with GFP-SKL(magenta)).

(e-g) Confocal microscopy of *A.thaliana* protoplasts (e) and roots (f-g) stained with BODIPY-C12 or PeroxiSPY650 (1µM, cyan) for 15min. GFP-SKL peroxisome marker visualized in magenta. Representative intensity profiles are shown.

Supplementary movie 1

Confocal microscopy of HeLa cells expressing GFP-SKL(magenta) were stained with PeroxiSPY650 (cyan), peroxisomes were visualized for 1hour. Scale bar 10µm.

Supplementary Figure 1

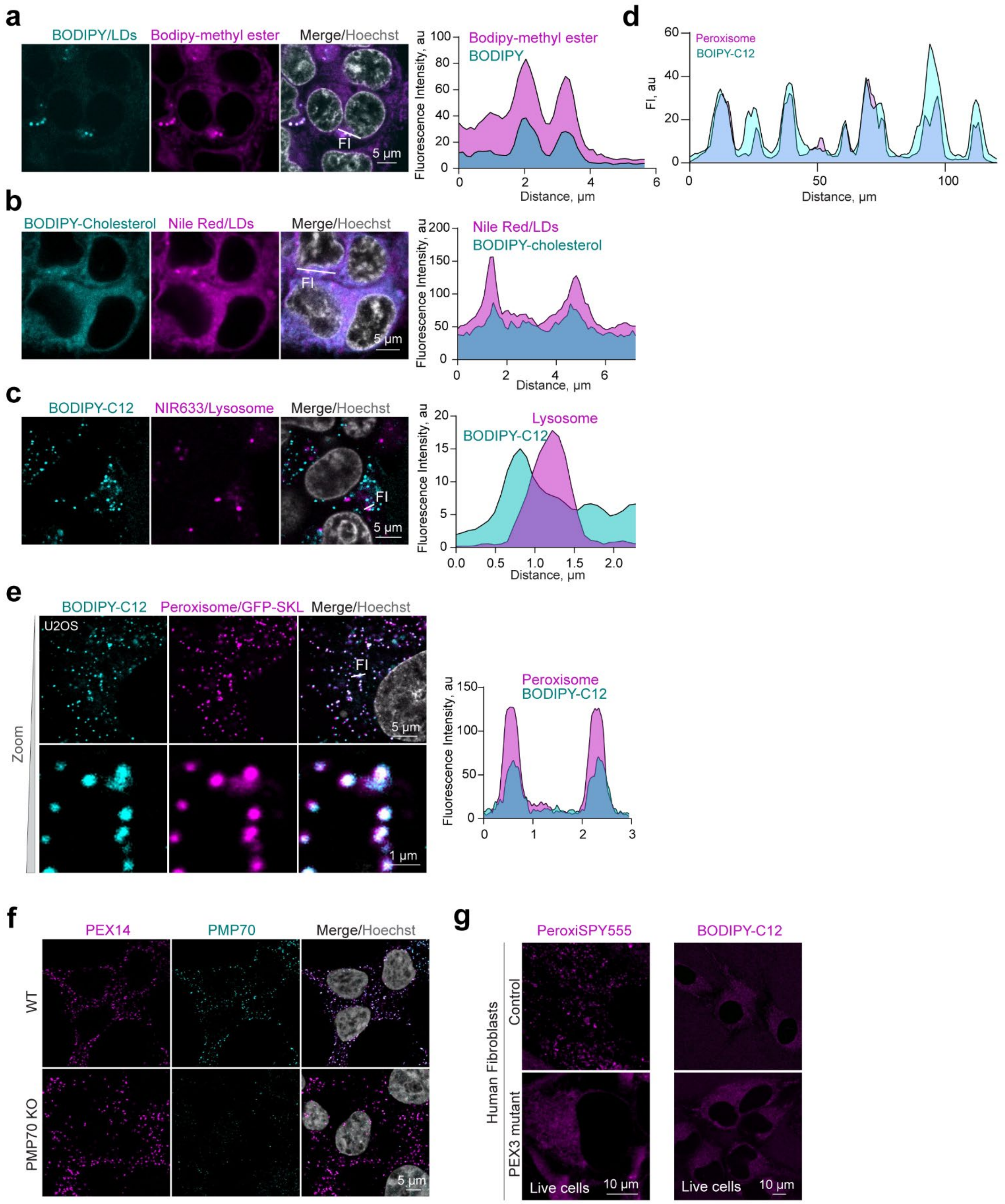

Supplementary Figure 2

a

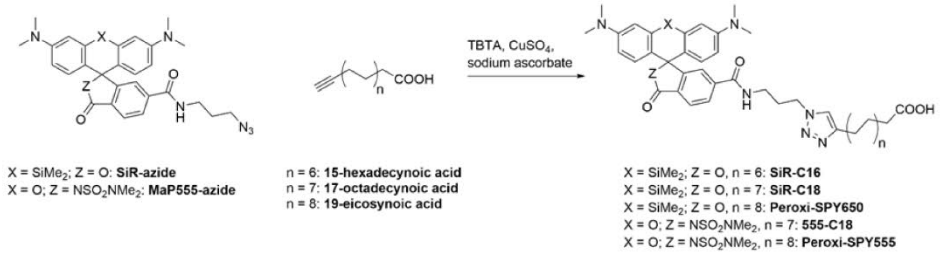

b

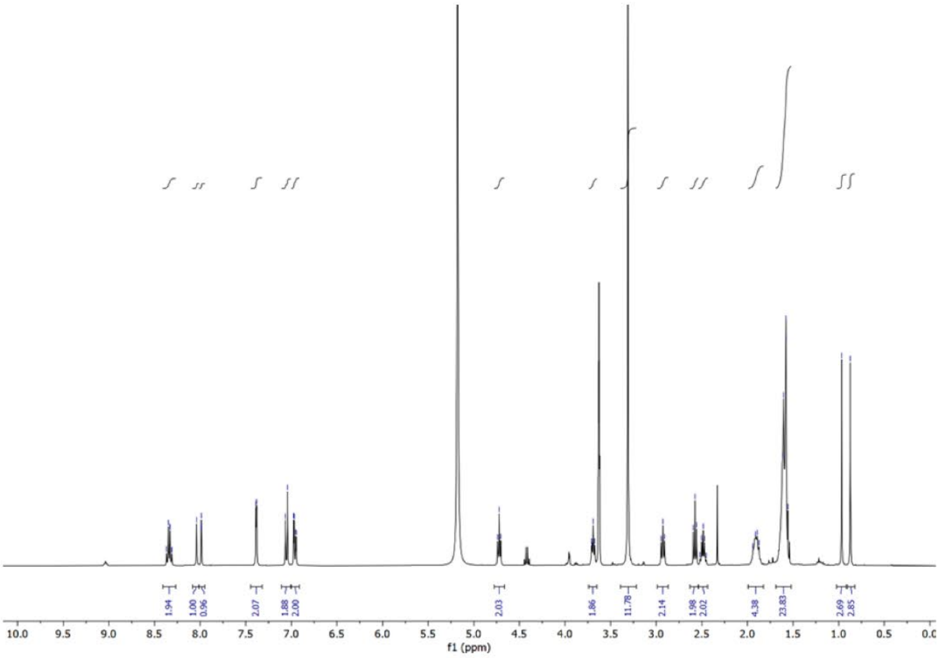

c

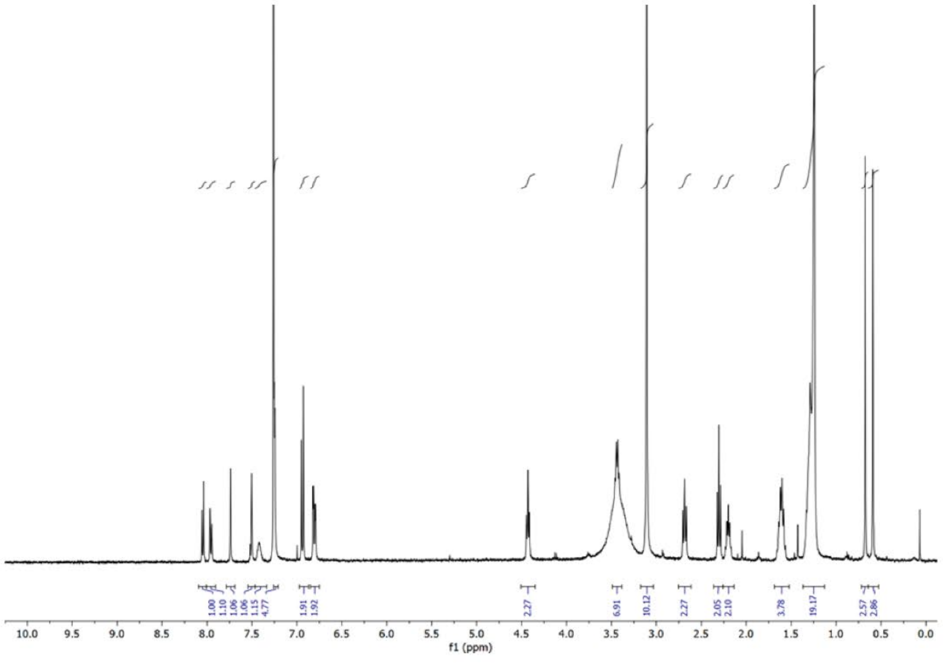

Supplementary Figure 3

a

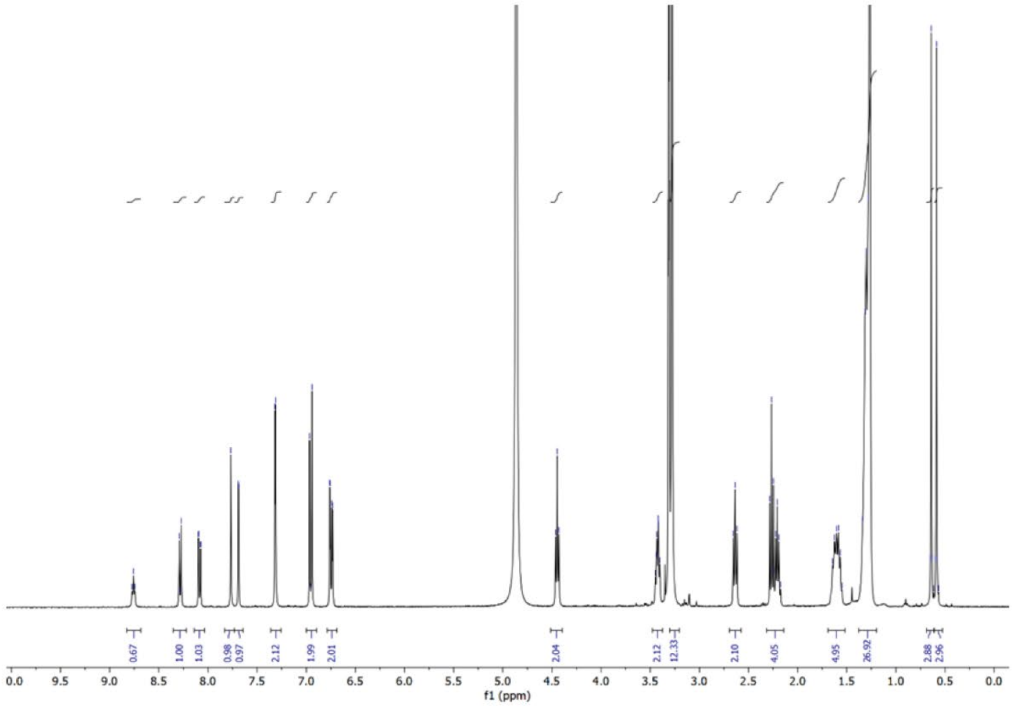

b

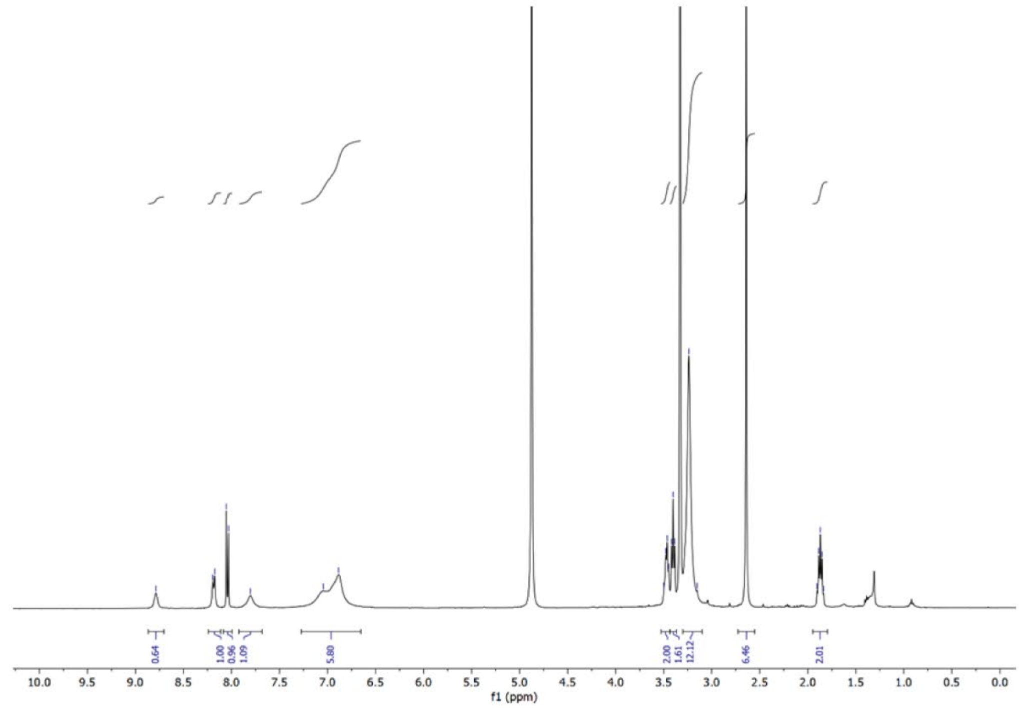

Supplementary Figure 4

a

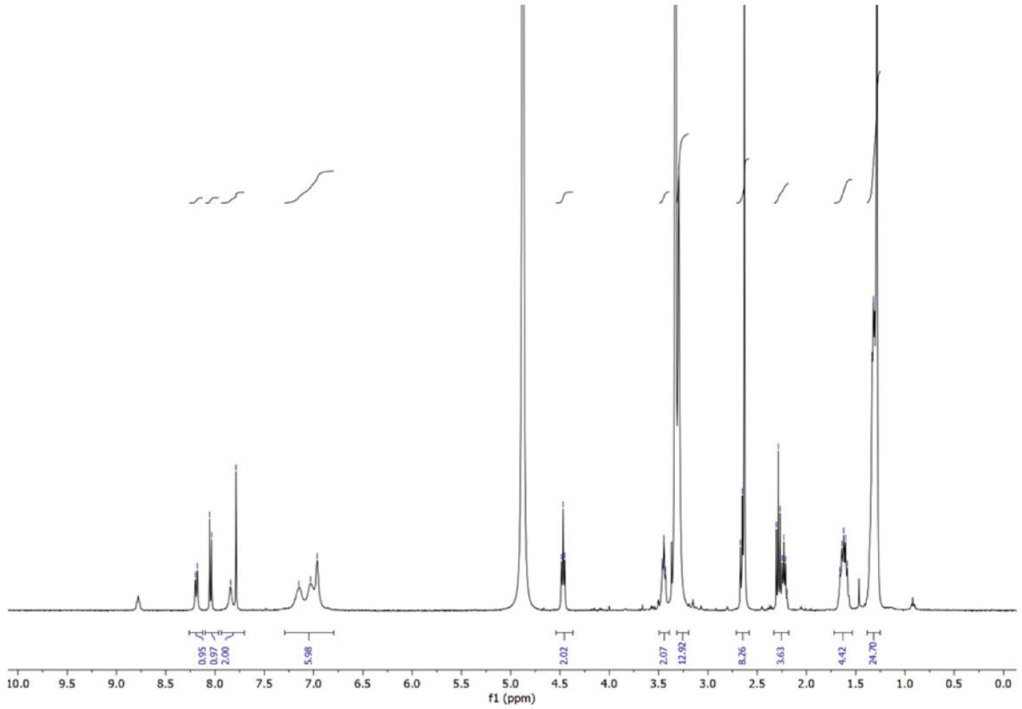

b

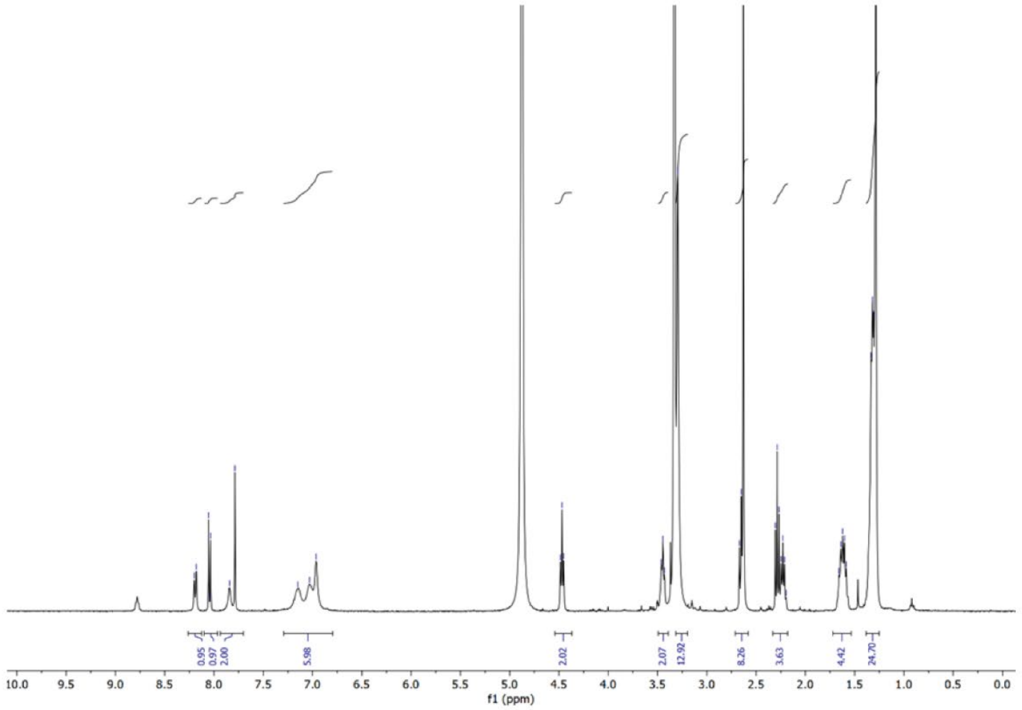

Supplementary Figure 5

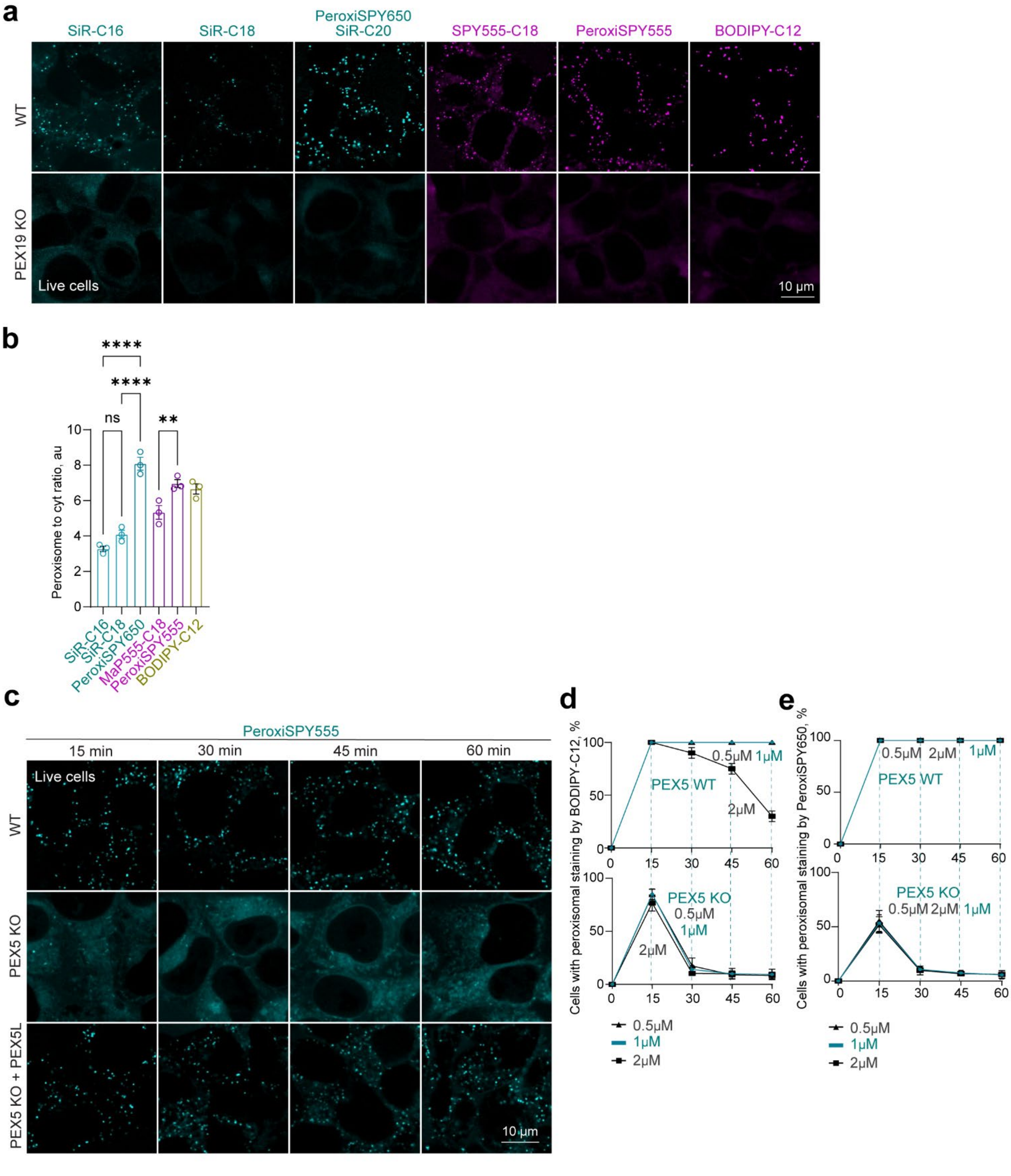

Supplementary Figure 6

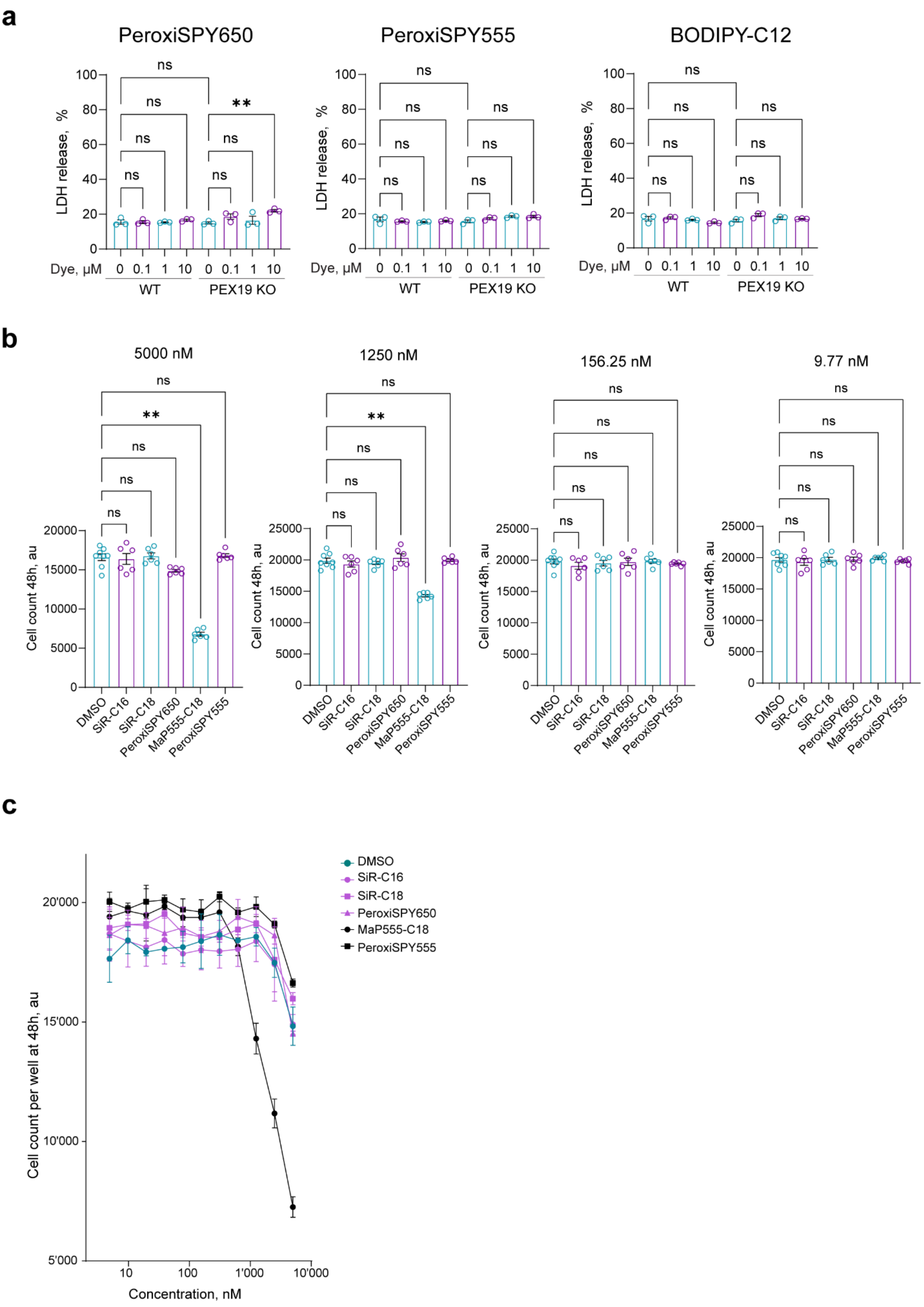

Supplementary Figure 7

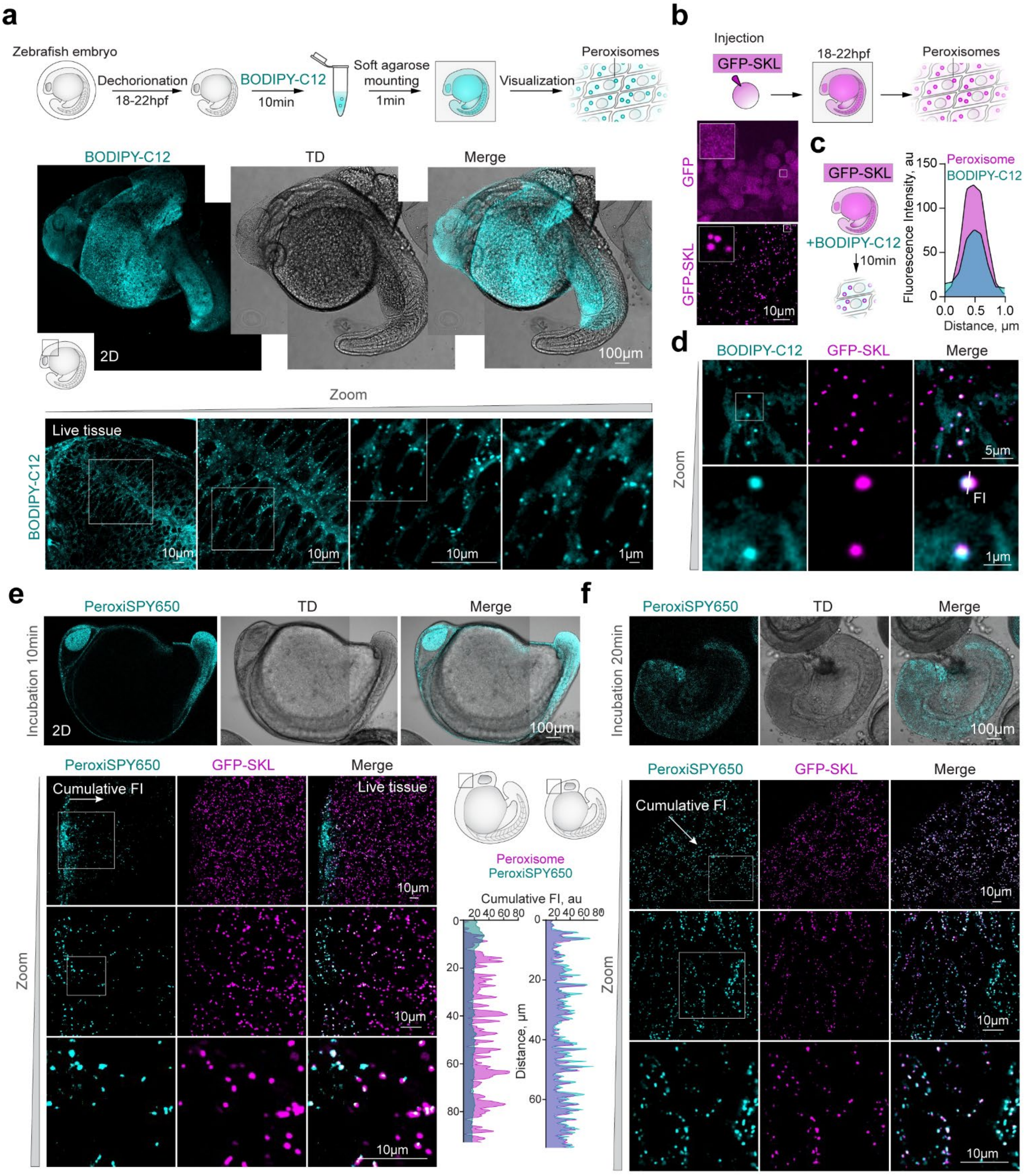

Supplementary Figure 8

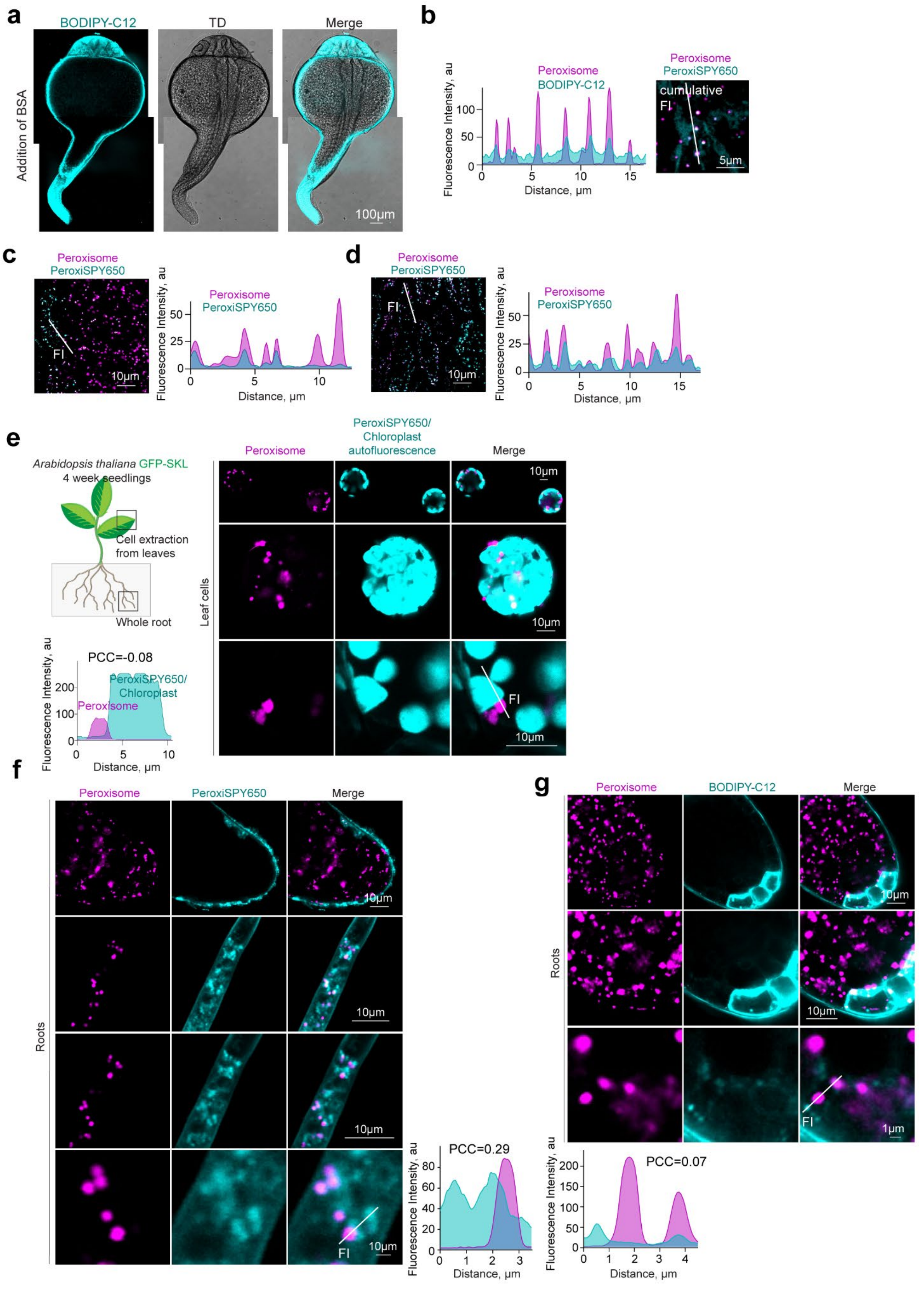

Supplement: Supplementary file 1 — Supplementary Information [file 41467_2024_48679_MOESM1_ESM.pdf]
