# Supplementary material for: Fluorescent fatty acid conjugates for live cell imaging of peroxisomes
Source: Nat Commun. 2024 May 21;15:4314. doi: 10.1038/s41467-024-48679-2 (PMC11109271; doi:10.1038/s41467-024-48679-2)
Supplement: Supplementary file 2 — Reporting Summary [file 41467_2024_48679_MOESM2_ESM.pdf]

# nature portfolio

Corresponding author(s): Triana Amen

Last updated by author(s): Apr 10, 2024

# Reporting Summary

Nature Portfolio wishes to improve the reproducibility of the work that we publish. This form provides structure for consistency and transparency in reporting. For further information on Nature Portfolio policies, see our Editorial Policies and the Editorial Policy Checklist.

## Statistics

For all statistical analyses, confirm that the following items are present in the figure legend, table legend, main text, or Methods section.

| n/a | Confirmed | |
|---|---|---|
| ☐ | ☒ | The exact sample size (*n*) for each experimental group/condition, given as a discrete number and unit of measurement |
| ☐ | ☒ | A statement on whether measurements were taken from distinct samples or whether the same sample was measured repeatedly |
| ☐ | ☒ | The statistical test(s) used AND whether they are one- or two-sided<br>*Only common tests should be described solely by name; describe more complex techniques in the Methods section.* |
| ☒ | ☐ | A description of all covariates tested |
| ☐ | ☒ | A description of any assumptions or corrections, such as tests of normality and adjustment for multiple comparisons |
| ☐ | ☒ | A full description of the statistical parameters including central tendency (e.g. means) or other basic estimates (e.g. regression coefficient) AND variation (e.g. standard deviation) or associated estimates of uncertainty (e.g. confidence intervals) |
| ☐ | ☒ | For null hypothesis testing, the test statistic (e.g. *F*, *t*, *r*) with confidence intervals, effect sizes, degrees of freedom and *P* value noted<br>*Give P values as exact values whenever suitable.* |
| ☒ | ☐ | For Bayesian analysis, information on the choice of priors and Markov chain Monte Carlo settings |
| ☒ | ☐ | For hierarchical and complex designs, identification of the appropriate level for tests and full reporting of outcomes |
| ☐ | ☒ | Estimates of effect sizes (e.g. Cohen's *d*, Pearson's *r*), indicating how they were calculated |

*Our web collection on statistics for biologists contains articles on many of the points above.*

## Software and code

Policy information about availability of computer code

| | |
|---|---|
| Data collection | LAS-X Leica SP8 software for images aquisition |
| Data analysis | FiJi/ImageJ for image analysis |

For manuscripts utilizing custom algorithms or software that are central to the research but not yet described in published literature, software must be made available to editors and reviewers. We strongly encourage code deposition in a community repository (e.g. GitHub). See the Nature Portfolio guidelines for submitting code & software for further information.

## Data

Policy information about availability of data

All manuscripts must include a data availability statement. This statement should provide the following information, where applicable:

- Accession codes, unique identifiers, or web links for publicly available datasets
- A description of any restrictions on data availability
- For clinical datasets or third party data, please ensure that the statement adheres to our policy

All data needed to evaluate the conclusions of the paper are present in the paper and supplementary materials. Source data are provided with this paper. All the reagents generated in this paper are available upon request from the corresponding author, TA. PeroxiSPY555 and PeroxiSPY650 will be commercially available at Spirochrome AG.

## Research involving human participants, their data, or biological material

Policy information about studies with human participants or human data. See also policy information about sex, gender (identity/presentation), and sexual orientation and race, ethnicity and racism.

| | |
|---|---|
| Reporting on sex and gender | n/a |
| Reporting on race, ethnicity, or other socially relevant groupings | n/a |
| Population characteristics | n/a |
| Recruitment | n/a |
| Ethics oversight | n/a |

Note that full information on the approval of the study protocol must also be provided in the manuscript.

# Field-specific reporting

Please select the one below that is the best fit for your research. If you are not sure, read the appropriate sections before making your selection.

[x] Life sciences [ ] Behavioural & social sciences [ ] Ecological, evolutionary & environmental sciences

For a reference copy of the document with all sections, see nature.com/documents/nr-reporting-summary-flat.pdf

# Life sciences study design

All studies must disclose on these points even when the disclosure is negative.

| | |
|---|---|
| Sample size | Sample sizes were not predetermined. Sample sizes were chosen based on the published literature on peroxisome quantification and analysis. The data and analysis were communicated to peroxisomal community during conference presentations and comments on data quantification were incorporated in the manuscript. |
| Data exclusions | No data were excluded from the analyses |
| Replication | At least three independent biological replicates were done for each experiment. All attempts at replication were successful. |
| Randomization | The fields for the microscopic analysis were randomly chosen using a Tile Scan of a nuclear staining on the confocal microscope. Organisms were randomly allocated into experimental groups. |
| Blinding | Blinding was not performed during data collection, due to a potential mixing of the samples with different genotypes. Group allocation during analysis was blinded. |

# Reporting for specific materials, systems and methods

We require information from authors about some types of materials, experimental systems and methods used in many studies. Here, indicate whether each material, system or method listed is relevant to your study. If you are not sure if a list item applies to your research, read the appropriate section before selecting a response.

## Materials & experimental systems

| n/a | Involved in the study | |
|---|---|---|
| [ ] | [x] | Antibodies |
| [ ] | [x] | Eukaryotic cell lines |
| [x] | [ ] | Palaeontology and archaeology |
| [ ] | [x] | Animals and other organisms |
| [x] | [ ] | Clinical data |
| [x] | [ ] | Dual use research of concern |
| [ ] | [x] | Plants |

## Methods

| n/a | Involved in the study | |
|---|---|---|
| [x] | [ ] | ChIP-seq |
| [x] | [ ] | Flow cytometry |
| [x] | [ ] | MRI-based neuroimaging |

## Antibodies

| | |
|---|---|
| Antibodies used | 1. anti-GAPDH, mouse monoclonal, sc-47724, Santa Cruz Biotechnology<br>2. anti-PMP70, mouse monoclonal, sab4200181, Sigma |

| | |
|---|---|
| | 3. anti-PEX19, rabbit polyclonal, 14713-1-AP, ProteinTech<br>4. anti-PEX5, rabbit polyclonal, hpa039260, Sigma<br>5. anti-VAPB, rabbit polyclonal, 14477-1-AP, ProteinTech<br>6. anti-PEX10, rabbit polyclonal, stj119242, St John's Laboratory<br>7. anti-VAPA, mouse monoclonal, sc-293278, Santa Cruz Biotechnology<br>8. anti-PEX14, rabbit polyclonal, 10594-1-AP, ProteinTech<br>9. Anti-Rabbit IgG Cy3-conjugated (Sigma-Aldrich C2306)<br>10. Anti-Mouse IgG Cy3-conjugated (Sigma-Aldrich C2181)<br>11. Anti-rabbit IgG Cy5 conjugated (Invitrogen A10523)<br>12. Anti-Mouse IgG H&L (Alexa Fluor® 488) (Abcam) |
| Validation | 1. https://www.scbt.com/p/gapdh-antibody-0411<br>2. https://www.sigmaaldrich.com/CH/en/product/sigma/sab4200181<br>3. https://www.ptglab.com/products/PEX19-Antibody-14713-1-AP.htm<br>4. https://www.sigmaaldrich.com/CH/en/product/sigma/hpa039260<br>5. https://www.ptglab.com/products/VAPB-Antibody-14477-1-AP.htm<br>6. https://stjohnslabs.com/anti-pex10-antibody-10-160-stj119242/<br>7. https://www.scbt.com/p/vap-a-antibody-4c12<br>8. https://www.ptglab.com/products/PEX14-Antibody-10594-1-AP.htm<br>9. https://www.sigmaaldrich.com/GB/en/product/sigma/c2306<br>10. https://www.sigmaaldrich.com/GB/en/product/sigma/c2181<br>11. https://www.thermofisher.com/antibody/product/Goat-anti-Rabbit-IgG-H-L-Cross-Adsorbed-Secondary-Antibody-Polyclonal/A10523<br>12. https://www.abcam.com/products/secondary-antibodies/goat-mouse-igg-hl-alexa-fluor-488-ab150113.html) |

## Eukaryotic cell lines

Policy information about cell lines and Sex and Gender in Research

| | |
|---|---|
| Cell line source(s) | 1. HeLa (a gift from Kaganovich Lab)<br>2. U2OS (a gift from Manley Lab, EPFL)<br>3. HEK293T (a gift from Kaganovich Lab)<br>4. Patient-derived iPSCs (control and proband)<br>5. Patient and control fibroblasts |
| Authentication | 1. Obtained from https://www.atcc.org/products/ccl-2 by Kaganovich Lab, no additional authentication was done.<br>2. Obtained from https://www.atcc.org/products/htb-96 by Manley Lab, no additional authentication was done.<br>3. Obtained from https://www.atcc.org/products/crl-3216 by Kaganovich Lab, no additional authentication was done.<br>4. The Genome Engineering & Stem Cell Center (GESC) at Washington University in St. Louis, Baylor College of Medicine, and Rarebase, PBC, peroxisome dysfunction was confirmed, no additional authentication was done.<br>5. Gift from Prof. Nancy Braverman (Canada), peroxisome mutations were confirmed, no additional authentication was done. |
| Mycoplasma contamination | All the cell lines tested negatively for mycoplasma contamination. |
| Commonly misidentified lines<br>(See ICLAC register) | No commonly misidentified cell lines were used in the study |

## Animals and other research organisms

Policy information about studies involving animals; ARRIVE guidelines recommended for reporting animal research, and Sex and Gender in Research

| | |
|---|---|
| Laboratory animals | Zebrafish Danio rerio, strain AB, 4-8 month old |
| Wild animals | No wild animals were used in the study |
| Reporting on sex | Gender analysis is not relevant to the study, no adult animals were used in the study, embryos under 24hpf were used. |
| Field-collected samples | No field-collected samples were used in the study |
| Ethics oversight | All fish were kept at the EPFL Zebrafish facility. All of the experiments were carried out using embryos derived from freely mating adults, and are therefore covered under the general animal experiment license of the EPFL granted by the Service de la Consommation et des Affaires Vétérinaires of the canton of Vaud—Switzerland (authorization number, VD-H23). Zebrafish embryos and larvae were used before they reached a protected developmental stage and require the authorization of the Ethics Committee. |

Note that full information on the approval of the study protocol must also be provided in the manuscript.

## Dual use research of concern

Policy information about dual use research of concern

### Hazards

Could the accidental, deliberate or reckless misuse of agents or technologies generated in the work, or the application of information presented in the manuscript, pose a threat to:

| No | Yes | |
|---|---|---|
| ☒ | ☐ | Public health |
| ☒ | ☐ | National security |
| ☒ | ☐ | Crops and/or livestock |
| ☒ | ☐ | Ecosystems |
| ☒ | ☐ | Any other significant area |

### Experiments of concern

Does the work involve any of these experiments of concern:

| No | Yes | |
|---|---|---|
| ☒ | ☐ | Demonstrate how to render a vaccine ineffective |
| ☒ | ☐ | Confer resistance to therapeutically useful antibiotics or antiviral agents |
| ☒ | ☐ | Enhance the virulence of a pathogen or render a nonpathogen virulent |
| ☒ | ☐ | Increase transmissibility of a pathogen |
| ☒ | ☐ | Alter the host range of a pathogen |
| ☒ | ☐ | Enable evasion of diagnostic/detection modalities |
| ☒ | ☐ | Enable the weaponization of a biological agent or toxin |
| ☒ | ☐ | Any other potentially harmful combination of experiments and agents |
