# Supplementary material for: Fluorescent fatty acid conjugates for live cell imaging of peroxisomes
Source: Nat Commun. 2024 May 21;15:4314. doi: 10.1038/s41467-024-48679-2 (PMC11109271; doi:10.1038/s41467-024-48679-2)
Supplement: Supplementary file 3 — Description of Additional Supplementary Files [file 41467_2024_48679_MOESM3_ESM.pdf]

Supplementary movie 1

Confocal microscopy of HeLa cells expressing GFP-SKL(magenta) were stained with PeroxiSPY650 (cyan), peroxisomes were visualized for 1hour. Scale bar 10µm.
